# Development and Validation of Performance-Based Assessment of Daily Living Tasks in Age-Related Macular Degeneration

**DOI:** 10.1167/tvst.13.6.9

**Published:** 2024-06-17

**Authors:** Anna C. S. Tan, Claire L. Peterson, Hla M. Htoon, Lynn L. Y. Tan, Yanwen Tan, Kai Ting Sim, Lisa Ong, Zhen K. Tan, Shih H. Heng, Ian Y. S. Yeo, Tien Y. Wong, Gemmy Cheung, Ryan Man, Eva K. Fenwick, Ecosse Lamoureux

**Affiliations:** 1 Singapore National Eye Centre, Singapore, Singapore; 2 Singapore Eye Research Centre, Singapore, Singapore; 3 Duke-NUS Medical School, Singapore, Singapore; 4 Singapore General Hospital, Singapore, Singapore; 5 University of Melbourne, Melbourne, Australia

**Keywords:** activities of daily living, age-related macular degeneration, outcome measures

## Abstract

**Purpose:**

To establish the reliability and validity of five performance-based activities of daily living task tests (ADLTT), to correlate structure to function, to evaluate the impact of visual impairment (VI) on age-related macular degeneration (AMD), and to develop new outcome measures.

**Methods:**

A multidisciplinary team developed five ADLTTs: (1) reading test (RT); (2) facial expression (FE) recognition; (3) item search (IS) task; (4) money counting (MC) task; and (5) making a drink (MD), tested with binocular and monocular vision. ADLTTs were tested for known-group (i.e., difference between AMD group and controls) and convergent (i.e., correlation to other measures of visual function), validity metrics, and test-retest reliability in 36 patients with VI (visual acuity (logMAR VA > 0.4) in at least one eye caused by AMD versus 36 healthy controls without VI.

**Results:**

Compared to controls, AMD patients had a slower reading speed (−77.41 words/min; *P* < 0.001); took longer to complete MC using monocular worse eye and binocular vision (15.13 seconds and 4.06 seconds longer compared to controls, respectively; *P* < 0.001); and MD using monocular worse eye vision (9.37 sec; *P* = 0.033), demonstrating known-group validity. Only RT and MC demonstrated convergent validity, showing correlations with VA, contrast sensitivity, and microperimetry testing. Moderate to good test-retest reliability was observed for MC and MD (interclass correlation coefficient = 0.55 and 0.77; *P* < 0.001) using monocular worse eye vision.

**Conclusions:**

Real-world ADL functioning associated with VI-related AMD can be assessed with our validated ADLTTs, particularly MC and MD.

**Translational Relevance:**

This study validates visual function outcome measures that are developed for use in future clinical practice and clinical trials.

## Introduction

Age-related macular degeneration (AMD) is the leading cause of irreversible visual impairment (VI) in older people.^1^^,^^2^ Outcomes in clinical trials have mostly focused on assessing visual function by use of visual acuity (VA). Studies in AMD patients have also shown that VA does not reflect the impact of VF loss on activities of daily living (ADL) function^3^^–^^8^ and the significant implications for the patient and their caregivers.

The assessment of ADL functioning is not routinely performed as an outcome measure in AMD in clinical practice,^9^ and in those that have, assessed ADL function used patient-reported outcome measures (PROMs).^3^^,^^5^ PROMs may be affected by the individual's disease perception, bias, and interpretation of the questions. Alternatively, patient-centered outcome measures (PCOMs), which are patient performance assessments that can be carried out in clinical settings, provide a more objective assessment of a patient's ADL function.^10^^–^^12^

PCOMs can also be tailored to focus on tasks that have been identified to be important and commonly performed by the target group of patients, such as reading ability in AMD.^13^^–^^15^ However, there are other PCOMs, apart from reading ability, that have been used in studies on elderly individuals, low vision populations, and in visual rehabilitation but not specifically in AMD populations.^10^^–^^12^^,^^16^^–^^20^ One of these scales, the Melbourne Low-Vision ADL Index has been shown in visually impaired patients to be a reliable, reproducible measure, with good correlation to other visual function measures and good responsiveness to visual rehabilitation.^18^^,^^21^ It consists of 18 performance based items (PCOMs) with more complex ADLs and nine self-reported items related to self-care ADLs.^18^ Another large study used PCOMs, based on mobility, daily living tasks, and visually intensive tasks such as facial recognition and reading speed that was also developed in 2001 showed that visually intensive tasks were better correlated with milder VI compared to mobility tasks.^20^ Previous timed instrument ADL performance tests have also been developed to test patients with moderate visual impairment from cataracts.^10^

During disease progression in AMD, early disease is associated with mild to moderate VI, progressing in an asymmetrical fashion in either eye to advanced diseases where central vision is affected resulting in central vision loss,^22^^–^^24^ leaving peripheral vision intact. Hence, we hypothesize that more visual-intensive, timed PCOMs, are needed to provide a more robust evaluation of real-life VF in patients with AMD when compared to rest of the VI population. Hence, we designed a series of ADL task tests (ADLTT) specifically for AMD patients that can be easily performed and measured in a clinical setting. The primary aim of this study is to determine the psychometric validity of the ADLTTs developed in a clinical sample of AMD patients and healthy controls using the guidance from the COnsensus-based standards for the selection of health Measurement INstruments (COSMIN) risk of bias checklist.^25^ We also aimed to use the validated ADLTTs as a measure to correlate retina structure and function and also as a potential outcome measure for future AMD studies.

## Methods

### Study Design and Setting

This was a cross-sectional study of patients >50 years with AMD and healthy controls, recruited from the outpatient clinic of a single tertiary ophthalmic center (Singapore National Eye Center, Singapore) from January 2017 to December 2020. The inclusion criterion for the AMD group was the diagnosis of AMD in at least one eye with VI for at least six months (logarithm of minimum angle of resolution (LogMAR ≥ 0.4) because of advanced AMD. Controls had no VI or eye disease and attended the retinal outpatient clinic for routine eye screening.

All participants were able to provide informed consent and had to have normal cognitive function as assessed by the Mini Mental State Exam,^26^ defined as a score of *>*25. Exclusion criteria for both groups included any significant media opacity preventing good quality imaging, any other eye disease that would significantly affect VA, and inability to perform functional tests because of a cognitive or physical disability. Physical disability was assessed via observation by the recruiting doctor, with recruited patients needing to have normal mobility, not wheelchair-bound, no obvious tremors or difficulty writing and have the appearance of full function of all their limbs. Ethical approval was obtained from the SingHealth Centralised Institutional Review Board (protocol number R1413/99/2016), and all investigations were conducted in accordance with the Declaration of Helsinki of 1975. Participants provided written informed consent before any study procedures were carried out.

### Study Assessments and Clinical Examination

All study subjects underwent a standard ophthalmic examination and measurement of monocular and binocular unaided visual acuity and best-corrected visual acuity (BCVA) using a LogMAR Chart, and contrast sensitivity (CS) was assessed with the Mars Perceptrix Contrast Test. Monocular microperimetry (MP-3, Nidek, Japan) was performed with a standardized protocol assessing mean retinal sensitivity (MRS) and mean macula sensitivity (MMS).

### Developing the ADLTTs

The ADLTT battery was developed specifically for this study, by an experienced multi-disciplinary group of low vision experts consisting of a group of ophthalmologists, optometrists and occupational therapists (A.T., L.T., K.S., L.O., Z.T., T.Y., J.H.). This team had daily consultations with VI patients with AMD and identified common ADLs that these patients faced. A thorough literature search was performed to identify other already existing PCOM studies.^18^^,^^20^ A survey in AMD patients and normal controls was conducted on how important they rate these specific ADLs, how often they perform the specific identified ADLs, and how they rate their ability to perform these tasks.

In comparison to the Melbourne Low-Vision ADL Index, which had 18 tasks, we had to compress and prioritize the number of tasks to be feasible for our study protocol. Ten out of the 18 tasks used reading ability, and this was compressed into reading a standardized text. Of the remaining tasks, our proposed ADLTTs included modified versions of the facial recognition, pouring, and identifying coins items. Of the remaining MVAI items, using a landline telephone and writing a bank check were less relevant in our modern society, and items such naming colors, buttoning a shirt, and threading a needle were less frequently performed by our target population compared to the latter tests and, hence, were excluded. We decided to increase the difficulty and complexity of the facial recognition, pouring and identifying coins items, and use the actual recorded timings required to complete the tasks for assessment measures similar to a previous study using timed ADLs(10). This was combined with qualitative ratings based on a Likert scale (0–3) on how independently the patient can complete each step of the tasks as assessed by the administrator (Supplementary Table S1). More detailed qualitative assessments of the individual steps taken to complete these tasks were also included as assessment measures for our proposed ADLTTs (Supplementary Table S1).

Mobility, although deemed important by our team, was a less visually intensive task,^20^ less significantly affected in AMD patients and was more difficult to perform, and posed the risks of falls; hence it was not included. Item search had been previously tested in both VI participants^16^ and also in AMD participants through monitor simulated scene^27^ and was chosen as an important ADLTT. Our team proposed a more real-life item search task, which was visually intensive, and based on the adaptation a visual search tasks used in patients with homonymous hemianopia.^28^

To perform structure-function correlations accurately, we had subjects perform the ADLTTs with both monocular and binocular vision. This is because binocular tests are not available for structural tests but ADL function is carried out with binocular vision. Each of our ADLTTs had to be performed at least twice to assess test-retest reliability, and with either eye occluded for monocular vision and then with binocular vision (3 sets of tests).

The specific components of the ADLTT are outlined below:

1. **ADLTT (1)- Reading Test (RT)** was assessed by reading speed and accuracy using the standardized International Reading Speed Texts (IReST)^29^ with the subject's own reading aids including the use of a standardized digital magnifier with known magnification. Illiterate subjects were exempt from this investigation but underwent other ADLTTs. This test has had previous validation and was available in both English and Mandarin.
2. **ADLTT (2)- Facial Expression recognition (FE)** was assessed based on the Warsaw set of emotional facial expressions, a set of high quality digital photographs presented to the subject.^30^ The number of correctly identified facial expressions and the time taken to identify each facial expression was recorded.
3. **ADLTT (3)- Item Search (IS)** included 16 standardized, everyday household items presented at a fixed distance, on pre-determined standardized position on the table equidistant apart chosen based on a previous publication.^28^ Both subject's eyes were first occluded and instructions were given to pick up four specified objects by the task administrator. The timing started from when the relevant eye/eyes were uncovered and the task was complete (timing stopped) when the subject had identified and picked up four items. The number of correct items identified and the time taken for the task completion was recorded.
4. **ADLTT (4)- Money Counting (MC)** assessed basic recognition of the denominations of local currency by presenting varying denominations of the local currency coins to the subject. The denominations were varied with each subsequent test to vary the total amount and to account for a learning effect, but there was a standardized total number of coins. The time taken to complete this task and the ability to complete each step of the task was graded (Supplementary Table S1).
5. **ADLTT (5)- Making a Drink (MD)** refers to the multi-step everyday standardized household task of making a drink. The steps include locating items, cup, and drink contents (sachets); pouring drink powder; pouring liquid; and stirring the drink. The time taken to complete this task and the ability to complete each step of the task was graded (Supplementary Table S1).

### Conducting the ADLTTs and Administrating Questionnaire

The study team underwent relevant ADLTT training and standardization specific for this study. Subjects performed all tests using BCVA with their best spectacle correction. At each visit, all subjects completed the five ADLTTs monocularly, where one eye was occluded with a patch, and binocularly, in a standardized room under 235 Lux (Amprobe LM-100 Light Meter) of light measured in the center of the room. If fatigue was observed by the administrator at any time, a rest break was given. The patient was also asked periodically if they needed a rest in between testing. Although monocular BCVA through occlusion created an artificial situation that deviates from a real world functioning, it had to be performed in addition to binocular testing to assess convergent validity, which required correlations to microperimetry, that can only be tested monocularly.

### Patient-Reported Outcome Questionnaire Assessment

The validated Impact of Vision Impairment (IVI) questionnaire with Rasch analysis using the Andrich rating scale model with Winsteps software (version 3.91.2),^31^ was used to measure VRQoL, with scores analyzed overall and in the individual domains of (1) Reading & Accessing Information, (2) Mobility & Independence, and (3) Emotional well-being.^2^^,^^31^^,^^32^

### Sample Size Calculations

Sample size calculations were estimated based on the expected mean difference of IReST text reading speed between participants with intermediate AMD (no VA cutoff) and healthy controls was 15 words/min (estimated SD 25).^33^ A larger mean difference in reading speed was assumed because of our worse VA criteria and more advanced AMD (20 words/min). Assuming a power of 0.80 and a significance of 0.05, giving a minimum sample size of 25 AMD cases and 25 normal controls, an additional 10 participants in each group was planned to improve potential statistical significance of our results for validity testing of the other ADLTTs.

### Validity Testing of the ADLTTs

We planned our validity testing based on a modified version of the COSMIN checklist^25^ using items of the checklist that were relevant to PCOM assessment (Appendix). The COSMIN checklist was originally developed for use to assess the quality and validity of patient-reported outcome measures; however, previous studies have applied the items of the COSMIN checklist to assess PCOMs in other diseases and contexts.^34^^,^^35^ Areas addressed by our validation study included PCOM development, content validity, cross-cultural validity, reliability and measurement error, convergent and known-group validity. Aspects of **PCOM development, content validity,** **and cross-cultural validity** have already been described above. The COSMIN risk of bias checklist only assessed whether the individual standards for psychometric properties were included in the validation process, not whether these psychometric properties were satisfactory.

All ADLTTs except RT (test-retest reliability already previously established^36^) were repeated twice (during the same session within three hours) consecutively to assess **test-retest reliability** and the average readings for each attempts were calculated. For tasks with continuous variable output (time), the interclass correlation coefficient (ICC) was used. For tasks that did not conform to continuous measured assumptions for repeatability such as “ability of tasks” and “number of items identified correctly,” Gwet's AC1 was used. For FE, which was an ordinal measure, a weighted Cohen's Kappa was used. Repeatability was graded as poor < 0.5, moderate = 0.5-0.75, good = 0.75-0.9, excellent > 0.9.^37^

**Convergent validity** was assessed by calculating the Spearman correlation coefficient to determine correlations between mean time taken to complete the various ADLTTs in AMD patients, with monocular and binocular BCVA, CS and monocular MRS and MMS. We hypothesised that all the five ADLTTs would demonstrate at least moderate (0.3 < ρ *>* 0.7) correlation (low correlation ≤ 0.3, high correlation ≥0.7).^38^

**Known-group validity** was assesed by comparing the ability grading and mean time taken to complete each ADLTT between AMD and control groups. All values were age-adjusted using analysis for covariance. Welch's *t*-test was used to test for significance of difference in means between the ADLTT groups. Our hypothesis was that significant differences in ADLTTs parameters would be observed between the AMD and control group.

For most of the ADLTTs proposed, no accepted gold standard exists hence criterion validity was unable to be tested. Responsiveness is assessed based on a longitudinal cohort and was not assessed in this current study, which only analyzes cross-sectional results.

### Statistical Analysis

Statistical analyses were performed using R studio Version 1.1.383 (RStudio, Inc. Boston, MA, USA) and SPSS (Released 2016. IBM SPSS Statistics for Windows, Version 24.0; IBM Corp, Armonk, NY, USA). STATA/SE 16.1 (Statscorp LLC) was used for GWET's AC repeatability testing. For all tests, *P* values <0.05 were deemed statistically significant. For divergent validity where discrete variables were correlated with continuous variables with the point-biserial correlation, which is mathematically equivalent to the Pearson (product moment) correlation was performed. We determined a cutoff value for poor ADLTT function based on a ninetieth percentile value of the normal healthy controls for quantitative variables such as reading speed, time taken to complete MC and MD. This models a similar analysis done in a previous publication.^39^ For the qualitative grading scores, we assumed that a healthy control would be able achieve a maximum score at each step of performing MC or MD (independent: experience no difficulty performing tasks safely, accurately and efficiently), hence anything less than the maximum score was deemed poorly functioning.

## Results

We recruited 36 AMD cases and 36 normal controls for this study. Baseline characteristics of AMD participants and healthy controls are presented in Supplementary Table S2. In the AMD group, out of the 36 pairs of eyes, 25 were pseudophakic and in the control group out of 36 pairs of eyes, 35 were pseudophakic.

Self-reported highest education levels for the AMD group versus the control group was primary education (12 [30%] vs. 8 [20%]), secondary education (19 [48%] vs. 16 [40%]), post-graduate degree and diploma and above (4 [10%] vs. 9 [23%]) (*P* = 0.3). Five AMD patients and seven controls refused to disclose this information.

Compared to controls, AMD participants were significantly older, had worse unaided visual acuity, BCVA and CS in the worse eye, (Supplementary Table S2). In the AMD group, out of the 36 patients with advanced AMD in at least one eye, four (11.1%) participants had bilateral VI (both eyes logMAR VA >0.4) from advanced AMD in both eyes. Out of all the eyes that had advanced AMD, four eyes had geographic atrophy whereas the rest had neovascular AMD as diagnosed on multimodal imaging. Sixteen additional AMD patients had the presence of intermediate or advanced AMD (Beckman classification) in the better vision eye with logMAR ≤0.4. In the AMD group, mean binocular VA, monocular VA in the better and worse eye was logMAR 0.17(±0.16), 0.23 (±0.20), and 1.01 (±0.60), respectively. In the AMD group, nine patients were unable to perform reading in at least one eye, eight patients were unable to perform FE successfully in one eye, two patients were unable to perform MC in one eye, and one patient was unable to perform MD in one eye. All patients in the AMD group were able to perform the IS task with either eye.

To assess content validity, based on the participant survey, MC and MD were reported to be performed daily and most frequently; and the majority of people described having to perform IS and FE at least once a month (Supplementary Figure S1). The mean rating of how important each ADLTTs was reported as above 7 (very- extremely important; 1 = not important, 10 = extremely important) for all five ADLTTs tested (Supplementary Table S3) in both AMD and controls. The mean self-reported ability of being able to perform each of these ADLTTs were reported as more than 8.5 for AMD and 10 for controls (mostly performed well with no help; 1 = cannot perform, 10 = performed well with no help) (Supplementary Table S3).

### Test-Retest Reliability of the ADLTT

There was poor repeatability for FE using monocular worse eye, better eye and binocular vision (Table 1). Moderate repeatability was observed in AMD participants for the number of items identified correctly in IS using monocular vision but poor using binocular vision (Table 1). Moderate repeatability was observed in the time taken to complete MC in AMD participants using their worse eye vision (Table 1). When performing MC, moderate repeatability was observed for AMD participants using the worse eye, using the better eye and binocular vision to perform MC showed poor repeatability (Table 1). Efficiency grading in completing MC showed moderate-good repeatability (Table 1). Good to excellent repeatability was observed for the time taken to complete MD for AMD participants using monocular worse or better eye and binocular vision (Table 1) and showed moderate to excellent repeatability of efficiency grading of this task (Table 1).

**Table 1.** Test-Retest Reliability of Performance-Based Measurement of the ADLTT in Persons With AMD Versus Normal Controls

Repeatability of ADLTT	Disease	*P* Value [95% CI]	Controls	*P* Value [95% CI]
Facial Expression^*^				
Mean number of expressions identified^†^				
Worse eye vision	0.08	−0.13: 0.27	0.25^‡^	0.01: 0.48
Better eye vision	0.15^‡^	−0.06: 0.38	0.20^‡^	−0.16: 0.26
Binocular vision	0.28	0.05: 0.50	0.29	0.08: 0.51
Item search task^§^				
Time taken to complete task				
Worse eye vision	0.55 (0.3–0.7)	<0.001	0.03 (−0.2–0.2)	0.63
Better eye vision	0.24 (−0.1–0.5)	0.07	0.16 (−0.2–0.5)	0.18
Binocular vision	0.43 (0.1–0.7)	<0.01	0.06 (−0.3–0.4)	0.37
Number of items identified correctly^‡^				
Worse eye vision	0.69 (0.5–0.9)	<0.001	0.77	<0.001
Better eye vision	0.72 (0.5–0.9)	<0.001	0.90 (0.8–1.0)	<0.001
Binocular vision (wt Kappa)	0.21	0.12	0.45	<0.01
Money counting task^†^				
Time taken to complete task				
Worse eye vision	0.55 (0.3–0.8)	<0.001	0.68 (0.6–0.8)	<0.001
Better eye vision	0.32 (0.6–1.9)	0.03	0.60 (0.3–0.8)	<0.001
Binocular vision	0.36 (0.1–0.6)	0.01	0.39 (0.1–0.6)	<0.01
Ability of task test completion^‡^				
Worse eye vision	0.54	<0.001	0.94	<0.001
Better eye vision	0.81 (0.6–0.9)	<0.001	1.00	NA
Binocular vision	0.88 (0.8–1.0)	<0.001	0.97 (0.9–1.0)	<0.001
Making drink^*^				
Time taken to complete task				
Worse eye vision	0.77 (0.6–0.9)	<0.001	0.92 (0.9–0.9)	<0.001
Better eye vision	0.91 (0.8–1.0)	<0.001	0.83 (0.7–0.9)	<0.001
Binocular vision	0.92 (0.9–1.0)	<0.001	0.92 (0.9–1.0)	<0.001
Ability of task test completion^‡^				
Worse eye vision	0.81 (0.7–0.9) ^‡^	<0.001	0.43 (0.3–0.9) ^§^	<0.001
Better eye vision	0.56 (0.2–0.9)^§^	<0.001	0.91 (0.8–1.0) ^‡^	<0.001
Binocular vision	0.97 (0.9–1.0) ^‡^	<0.001	0.97 (0.9–1.0) ^‡^	<0.001

For tasks with continuous variable output (Time), Interclass Correlation was used.

* Mean score taken over three attempts.

† For Facial expression, weighted Cohen's Kappa was used with a 95% confidence interval instead of a *P* value displayed.

‡ For tasks with categorical values (Efficiency of tasks and Number of items identified correctly), Gwet's AC −1 was used. Repeatability: poor = <0.5, moderate = 0.5-0.75, good = 0.75-0.9, excellent >0.9.

§ Mean values taken over two attempts.

### Assessment of Convergent Validity of the ADLTT

In the AMD group, convergent validity was observed for RT, with a moderate-strong correlation of worse reading speed with worse monocular BCVA (Spearman correlation coefficient (ρ) = −0.74; *P* < 0.001) and binocular BCVA (ρ = −0.44; *P* = 0.01) (Table 2). A moderate-strong better reading speed correlation with better monocular CS (ρ = 0.59; *P* < 0.001), and higher MRS (ρ = 0.56; *P* < 0.001) was also observed (Table 2). For MC, there was a moderate-strong longer time taken to complete the task with worser binocular BCVA (ρ = 0.34; *P* = 0.045) and monocular BCVA (ρ = 0.71; *P* < 0.001)(Table 2). A moderate-strong correlation with longer time to complete test and poorer monocular CS (ρ = −0.62; *P* < 0.001) and lower MRS (ρ = −0.56; *P* < 0.001) was also observed (Table 2). No significant correlations were observed with FE, IS or MD (*P* > 0.05). No correlations with the IVI score (VF domain) were observed with ADLTT performed with either monocular or binocular vision (Supplementary Table S4).

**Table 2.** The Assessment of Convergent Validity in the Age-Related Macular Degeneration Group Though the Spearman Correlation of Binocular and Monocular Performance Based on ADLTT With Other Conventional Measures of Visual Function

	Binocular				Monocular							
Task	BCVA	*P* Value	CS	*P* Value	BCVA^*^	*P* Value	CS^*^	*P* Value	MRS^*^	*P* Value	MMS^*^	*P* Value
Reading speed (words/min)	−0.44	0.01	0.22	0.21	−0.74	<0.001	0.59	<0.001	0.56	<0.001	−0.11	0.40
Number of facial expressions identified	−0.22	0.22	−0.16	0.35	−0.12	0.40	0.11	0.37	0.08	0.55	0.16	0.20
Time taken to complete the item search task (s)	−0.06	0.74	−0.04	0.84	−0.11	0.35	−0.18	0.14	0.15	0.21	−0.08	0.51
Time taken to complete the money counting task (s)	0.34	0.05	−0.30	0.08	0.71	<0.001	−0.62	<0.001	−0.56	<0.001	0.10	0.40
Time taken to complete the making drink task (s)	0.06	0.74	−0.08	0.63	0.01	0.92	<−0.01	0.97	−0.10	0.42	−0.02	0.90

Reference Chan YH. Biostatistics 104: Correlational Analysis. *Singapore Med J*. 2003;44:614–619.

* Significant *P* value < 0.05 refers to monocular values from both the better and worse eyes analysed together for the correlation analysis. Spearman correlation coefficient categories: poor = <0.3, fair = 0.3-0.59, moderately strong = 0.6-0.79, very strong 0.80-1.0

### Assessment of Known-Group Validity of the ADLTT

#### RT

Using monocular BCVA (worse eye), AMD participants had a significantly slower reading speed (−77.41 words/min [*P* < 0.001]) and less correct words (−11.54 words [*P* < 0.001]) compared to controls (Table 3; Figure), suggesting a good known-group validity for this test.

**Table 3.** The Assessment of Known-Group Validity in Performance-Based Measurement of the ADLTT in Persons With AMD Versus Normal Controls Adjusted for Age^*^

ADLTT	AMD (n = 36)	Controls (n = 36)	*P* Value^†^
Reading tests			
Speed (words/min ± SD)			
Worse eye vision	62.59 ± 42.6	140.00 ± 42.0	**<0.001**
Better eye vision	132.34 ± 40.8	142.51 ± 40.8	0.33
Binocular vision	136.46 ± 41.4	147.62 ± 42.6	0.31
Facial expression^‡^			
Mean number of expressions identified (max = 5 ± SD)			
Worse eye vision	3.23 ± 0.9	3.42 ± 0.8	0.38
Better eye vision	3.44 ± 0.7	3.53 ± 0.7	0.58
Binocular vision	3.76 ± 0.8	3.67 ± 0.8	0.68
Item search task^§^			
Time taken to complete task (s ± SD)			
Worse eye vision	11.54 ± 7.2	9.69 ± 7.6	0.31
Better eye vision	8.64 ± 3.8	8.29 ± 3.8	0.71
Binocular vision	7.37 ± 4.0	7.59 ± 4.0	0.83
Number of items identified correctly (max = 4 ± SD)			
Worse eye vision	3.78 ± 0.4	3.77 ± 0.4	0.95
Better eye vision	3.90 ± 0.3	3.83 ± 0.3	0.31
Binocular vision	3.60 ± 0.4	3.50 ± 0.4	0.98
Money counting task^§^			
Time taken to complete task (s ± SD)			
Worse eye vision	21.39 ± 11.7	6.26 ± 11.7	**<0.001**
Better eye vision	9.41 ± 7.2	5.97 ± 7.0	0.06
Binocular vision	8.14 ± 4.3	4.08 ± 4.3	**<0.001**
Ability of task completion (max score = 9 ± SD)			
Worse eye vision	7.54 ± 1.4	8.89 ± 1.4	**<0.001**
Better eye vision	8.61 ± 0.6	8.98 ± 0.6	**0.02**
Binocular vision	8.91 ± 0.3	8.96 ± 0.2	0.43
Making drink task^§^			
Time taken to complete task (s ± SD)			
Worse eye vision	53.99 ± 17.2	44.62 ± 17.5	**0.03**
Better eye vision	51.50 ± 17.7	44.00 ± 16.9	0.09
Binocular vision	48.02 ± 19.7	40.98 ± 19.7	0.16
Ability of test completion (max score = 15 ± SD)			
Worse eye vision	14.24 ± 1.1	14.79 ± 1.1	**<0.05**
Better eye vision	14.71 ± 0.9	14.75 ± 0.9	0.87
Binocular vision	14.93 ± 0.3	14.98 ± 0.3	0.43

SD, standard deviation.

* Analysis for covariance used to adjust for impact of age on means.

† *P* value calculated by Welch's *t*-test for quantitative variables.

^‡^Mean score taken over three attempts.

§ Mean values taken over two attempts.

**Figure.**
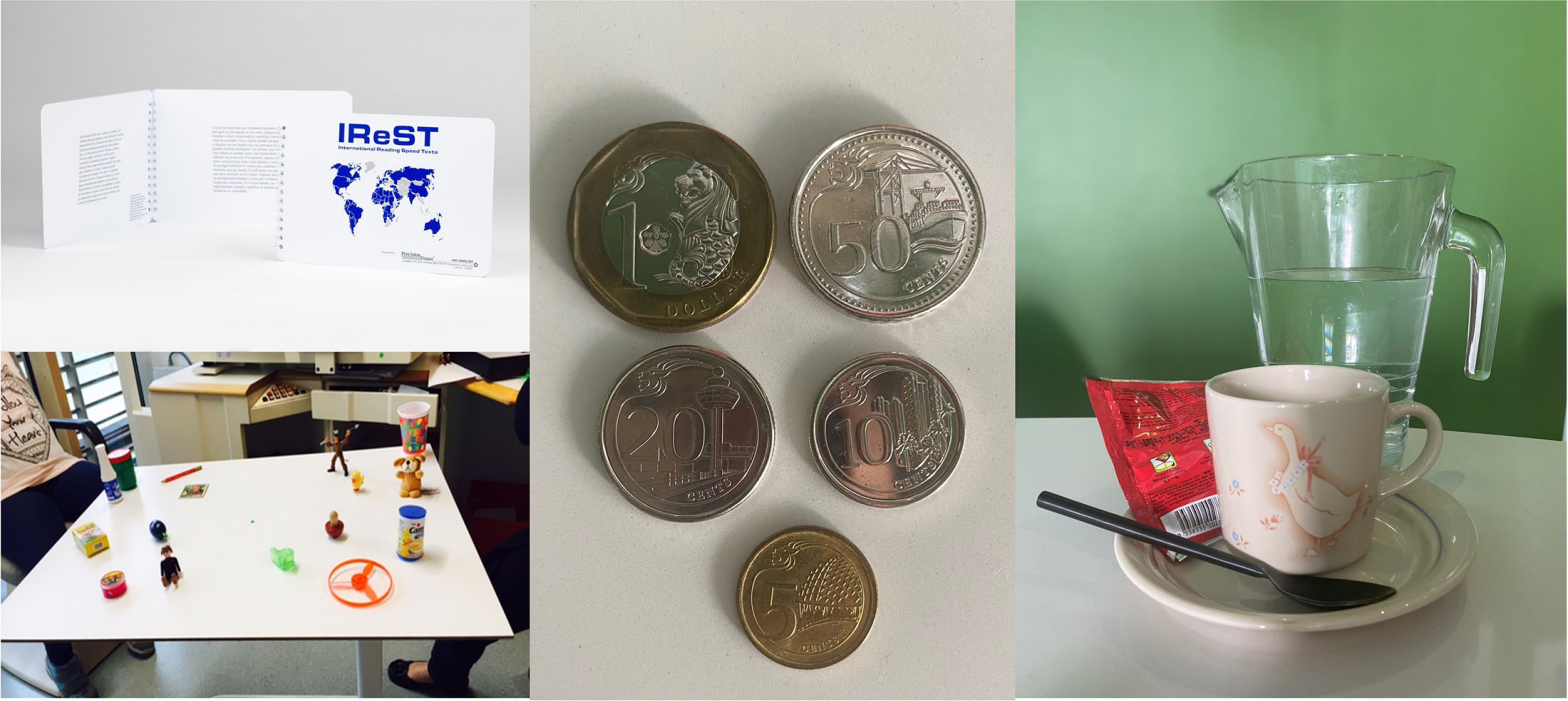
Representation pictures of activities of daily living tasks test include the IReST reading charts (top left), the set-up of the item search task layout (bottom left), local currency coins used in different combinations for the assessment of money counting (middle) and the various components used in the making a drink task (right).

#### FE

There was no statistically significant difference between AMD participants and healthy controls in the mean number of expressions identified correctly, using either monocular or binocular BCVA, showing poor known-group validity.

#### IS

There was no statistically significant difference between AMD participants and healthy controls for the time taken to complete the task and the number of items using either monocular or binocular BCVA identified correctly, showing poor known-group validity (Table 3; Figure).

#### MC

Using both monocular (worse eye) and binocular BCVA, AMD participants required significantly longer to complete the money counting task (15.13 sec [*P* < 0.001]) and (4.06 sec [*P* < 0.001]), respectively, compared to controls Table 3; Figure). Ability scores for this task were significantly worse for AMD participants versus controls using both the worse eye (−1.35 [*P* < 0.001]) and better eye (−0.37 [*P* = 0.016]) BCVA. These findings suggests excellent known-group validity.

#### MD

Using monocular worse eye BCVA, AMD participants took significantly longer to complete the multi-step task (9.37 sec [*P* = 0.033]) and had a lower ability score (−0.55 sec [*P* = 0.048]), respectively, compared to controls Table 3; Figure), suggesting excellent known-group validity.

Known-group validity based on VA regardless of AMD status was also assessed (Supplementary Table S5). Similar trends supportive of known-group validity were observed for both monocular and binocular RT and MC (time taken), as well monocular MD (time taken), especially for those with VA >0.7 LogMAR. However, these differences were overall not statistically significant due to small numbers of those with poor VA.

Table 4 provides a summary of the various psychometric properties of the 5 ADLTTs. We have established the normal cut-off ranges for the quantitative variable of the validated ADLTTs (RT, MC and MD) based on the normal range ninetieth percentile cutoff and assumed a qualitative ability score of less than the maximum score as poor function (Table 5). The overall development and validation of our proposed PCOMS based on the modified COSMIN checklist was assessed in Supplementary Table S6.

**Table 4.** Summary of Validation Results and Test-Retest Reliability Results of ADLTTs

ADLTT	Known Group Validity	Convergence Validity to Other Visual Function Measurements	Test-Retest Reliability^*^
Reading tests			
Speed (words/min)			
Worse eye vision	Yes	Moderately negative: mBCVA	N/A
Better eye vision	No	Fair: mCS, MRS	N/A
Binocular vision	No	Fair: biBCVA	N/A
Facial Expression			
Mean number of expressions identified (max = 5)			
Worse eye vision	No	No significant correlation	Poor
Better eye vision	No		Poor
Binocular vision	No		Poor
Item search task			
Time taken to complete task (s ± SD)			
Worse eye vision	No	No significant correlation	Moderate
Better eye vision	No		Poor
Binocular vision	No		Poor
Number of items identified correctly (max = 4)			
Worse eye vision	No	N/A	Moderate
Better eye vision	No	N/A	Moderate
Binocular vision	No	N/A	Poor
Money counting task			
Time taken to complete task (s ± SD)			
Worse eye vision	Yes	Moderate positive: mBCVA, Moderate negative: mCS Fair: biBCVA, Fair: MRS	Moderate
Better eye vision	No		Poor
Binocular vision	Yes		Poor
Efficiency of task test completion (max score = 9)			
Worse eye vision	Yes	N/A	Moderate
Better eye vision	Yes	N/A	Good
Binocular vision	No	N/A	Good
Making drink task			
Time taken to complete task (s ± SD)			
Worse eye vision	Yes	No significant correlation	Good
Better eye vision	No		Excellent
Binocular vision	No		Excellent
Efficiency of task test completion (max score = 15)			
Worse eye vision	Yes	N/A	Good
Better eye vision	No	N/A	Moderate
Binocular vision	No	N/A	Excellent

M, monocular; Bi, binocular.

Reference Chan YH. Biostatistics 104: Correlational Analysis. *Singapore Med J*. 2003;44:614–619.

* ICC: poor <0.5, moderate = 0.5-0.75, good = 0.75-0.9, excellent >0.9. Spearman correlation coefficient categories: poor <0.3, fair = 0.3-0.59, moderately strong = 0.6-0.79, very strong 0.80-1.0

**Table 5.** Recommended Grading of validated ADLTT for AMD Patients, Reading, Money Counting, and Making a Drink

	Poor	Acceptable
Reading test (IREST)		
Speed (words/min)^*^	<102	≥102
Money counting tasks		
Time taken to complete task (seconds)^*^	>11	≤11
Ability of task completion (max 9)	<9	9
Making drink tasks		
Time taken to complete task (seconds)^*^	>59	≤59
Ability of task completion (max 15)	<15	15

* Values calculated based on 90% percentile of healthy control values for reading speed, time taken for MC and MD, rounded to nearest number, the cut-off for poor qualitative ability grading is less than the maximum (see Supplementary Fig. S2).

## Discussion

Three out of our five ADLTTs, RT, MC and MD, demonstrated satisfactory age-adjusted known-group validity; and reasonable test-retest repeatability and have the potential to be included in routine clinical practice and future research studies as PCOMs of visual functioning in AMD patients. In particular RT and MC showed good convergent validity in the AMD group, with moderate-high correlations with other related measures of VF, such as VA, contrast and MRS on microperimetry testing. Of note, some weaker correlations with VF measurements were observed with RT, IS and MD in the control group compared to the AMD group, and the significance of this is uncertain. Selected ADLTTs that performed well in this validation study, such as RT, MC and MD, are tasks that are performed commonly by AMD participants, and can be replicated in a clinical setting without the need for extensive resources. In addition, these specific ADLTTs are rated as important both AMD patients and controls in our study (Supplementary Fig. S1). Based on our results, we determined that the psychometric properties of RT, M,C and MD to be satisfactory. We proposed cutoff values based on our study results for suboptimal ADLTT function (Supplementary Fig. S2, Table 5) as a basis for future studies, which may further validate these results in a cohort of patients with less advanced AMD.

In our AMD patients, self-reported rating on their ability to complete these tasks were accurate, and the high qualitative grading scores showed that most AMD patients were able to complete the ADLTTs (Supplementary Table S3). Our proposed validated ADLTTs such as RT, MD and MC have the potential to be applied to other types of macula disease but will require further validation studies. At this point in time, based on our validation study, ADLTTs are to be scored separately and provide users with separate, specific information that may be used for directed visual rehabilitation training. At this stage, no overall score is recommended for the ADLTT because some of the ADLTTs need refining; however, future research will endeavor to fit the completion scores to the Rasch model using the Method of Successive Dichotomizations,^40^ which will allow an overall ADL score to be reported.

Our study showed that FE and IS had poorer psychometric properties with no known-group validity, convergent validity, and poor test-retest repeatability and are currently not suitable for use in clinical practice. We are currently reviewing protocols and troubleshooting these ADLTTs in an effort to further improve repeatability during follow up ADL testing. FE was based on a standardized facial expression scale designed for use to identify particular emotions and based on a set of photographs of Western faces.^30^ In our local context, some of these emotional expressions were unfamiliar to participants, with limited understanding of the subtleties between some emotions (e.g., fear vs. disgust). Hence, we are currently developing an improved simplified facial expression identification scale with culturally applicable faces to measure facial expression recognition suitable for use in Asian participants who have VI. Future work is still needed to examine criterion, convergent and known-group validity and test-rest reliability for our revised FE tasks to be included in our ADLTT battery. For IS, we have attempted to improve repeatability by giving some practice attempts to perform the motions of these tasks before the actual results are recorded to give participants better familiarity with the basic motions of the tasks and also specifying the positions of each of the IS items on each attempt. In addition, IS was previously adapted from a test designed for use in a population with hemianopia and it may not represent a real world scenario or may be too simple in the AMD population where VI may not be as severe. Possible ways that we could increase the difficulty of the search task, would be to simulate more real-world situations (e.g., “cluttered table” or “a bookshelf with multiple objects”). Adding the eye tracking as an outcome measure during the search tasks would be also useful to understand the visual movements required to perform these tasks. Using computerized testing to create real-world scenarios and using wearable headsets that can monitor eye movements are possible research possibilities for the future. However, based on the lower frequency of how often participants report performing these 2 ADLTTs (Supplementary Fig. S1), it may also be reasonable to exclude these tasks in future studies to focus on more common tasks with better psychometric properties.

Although none of the ADLTT correlated with the specific domains of the IVI, this may be because this is a relatively small sample size to measure PROMs and the ADLTT is a performance assessment that measures real-life ADL functioning, which may not always be consistent with patient-reported effects of VF, making it suitable as an alternative outcome measure. We did not observe any differences in scores for RT, FE and IS between the AMD and control groups when using binocular vision, which may be explained by the fact that in the AMD group, only 4 (11.1%) participants had bilateral visual impairment (both eyes logMAR VA >0.4). Hence, for certain ADLTTs, the difference in the ability to perform ADLTT between AMD and control groups may have been diminished as the majority of participants could still rely on their better eye vision, especially in our participants where often the better eye vision is similar to the binocular vision. In contrast, the MC test did show good known-group validity using binocular vision, which is likely because these were more complex tasks requiring multiple steps that rely more on binocular VF.^41^^–^^44^ We acknowledge that it would have been preferable to include a larger sample of patients with binocular AMD and VI. However, these patients are less common and often elderly with co-morbidities, and therefore less willing to participate in physically and time demanding studies.

The strength of this study is that it uses a robust case-control study design with standardized protocols with trained research staff to assess the ADLTTs and other functional assessments of vision, including contrast sensitivity and microperimetry testing, which are often lacking in other validation studies. Moreover, we assessed both better and worse eye monocular, and binocular vision, allowing correlations with monocular VF measurements such as microperimetry. We also acknowledge that this rigorous protocol may have resulted in some fatigue for patients during the session, hence efforts were made to give them frequent breaks and no obvious deterioration of ADLTT function was noted by administrators during repeated testing in a single session. Because of the lack of previous data on these specific ADLTTs, a limitation of this study is the small sample size, meaning that it may be underpowered to show known-group validity for certain ADLTTs, and we are currently collecting a larger cohort for longitudinal testing. Another limitation of this study is that more subtle disabilities, not obvious to the observer and unrelated to their vision, may not have been accounted for in this study. We also restricted the assessment of reading ability to the IRest standardized reading text, and we acknowledge this may not fully reflect other reading tasks performed in a real-world setting such as reading medication labels or food packaging. However, because of time limitations and the need for reading tasks to be conducted in both English and Chinese, the IRest test was deemed the most suitable. We also acknowledge that in this validation study, our proposed ADLTTs were designed for our local context, and for these performance assessments to be more widely implemented, further validation and implementation studies with opinions and consensus from ophthalmologists, allied health specialists, and patients from more diverse backgrounds is needed. For example, MC is largely dependent on the appearance of the currency denominations, and this may vary in color and size, which may affect the difficulty of this tasks.

In conclusion, three of our objectively measured series of ADLTTs (RT, MC and MD) displayed excellent validity and reliability results in patients with VI due to AMD. Although satisfactory psychometric properties were observed most commonly in worse vision eyes, it was encouraging that MC using the better vision eye and binocular vision also showed known-group validity, hence can be considered for use in the assessment of individuals with mild VI. Further testing of protocols, that may increase the difficulties of RT and MD may have the potential to show known group validity even in cases with milder VI. Further work is required to refine the remaining ADLTTs, to enable us to develop valid, reliable and objective PCOM assessments, which can complement current PROMs, to provide a holistic assessment of patients’ real world VF. These measures have important applications for clinical practice, clinical trials, and are relevant to funding bodies and health authorities for assessing value-based healthcare.

## Supplementary Material

Supplement 1

Supplement 2

Supplement 3

Supplement 4

Supplement 5

Supplement 6

Supplement 7

Supplement 8

Supplement 9

## Appendix 1 Appendix. Modified COSMIN checklist for the assessment of PCOMs

Validated performance based standardized ADL task tests are complimentary clinical outcome measures of real-life functioning in patients with AMD (See Supplementary File S9).
