# Supplementary material for: Development and Validation of Performance-Based Assessment of Daily Living Tasks in Age-Related Macular Degeneration
Source: Transl Vis Sci Technol. 2024 Jun 17;13(6):9. doi: 10.1167/tvst.13.6.9 (PMC11185266; doi:10.1167/tvst.13.6.9)

Supplementary figure 1 : The self-reported frequency of performing the various acitvity of daily living tasks tests

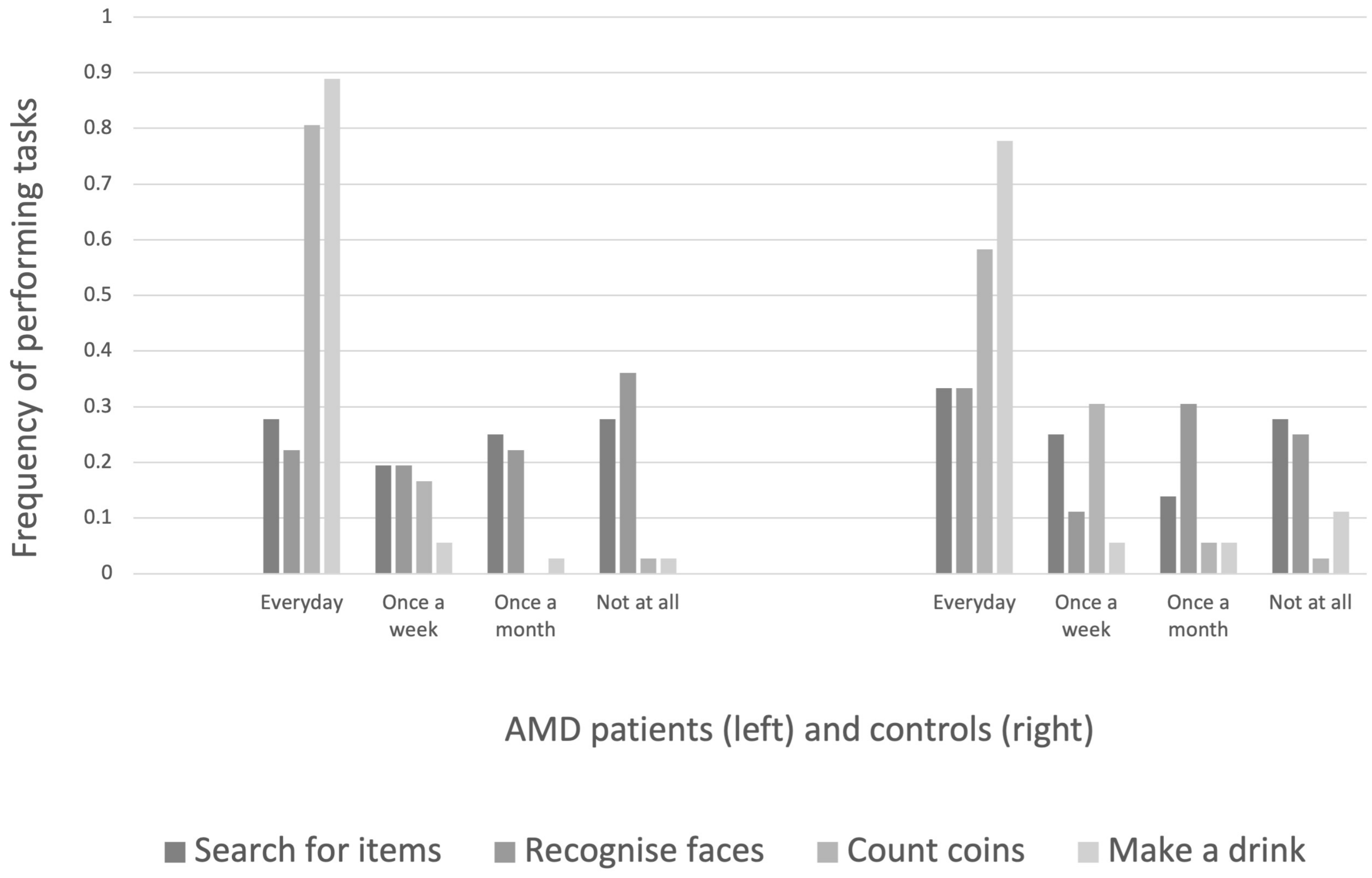

Supplement: Supplement 1 [file tvst-13-6-9_s001.pdf]
