# Supplementary figures and images for: Development and Validation of Performance-Based Assessment of Daily Living Tasks in Age-Related Macular Degeneration
Source: Transl Vis Sci Technol. 2024 Jun 17;13(6):9. doi: 10.1167/tvst.13.6.9 (PMC11185266; doi:10.1167/tvst.13.6.9)

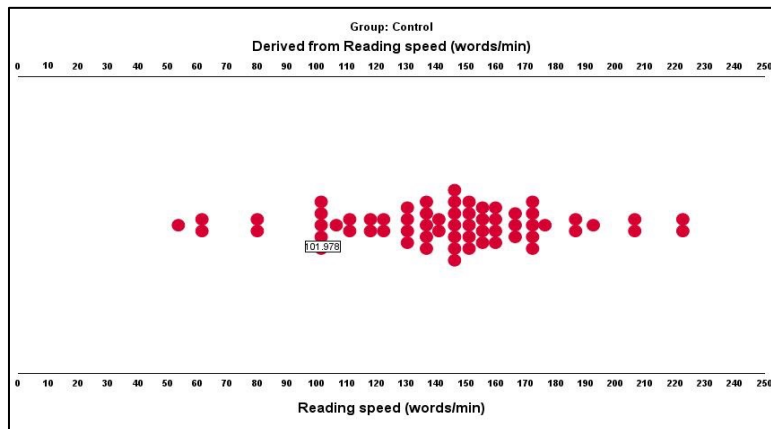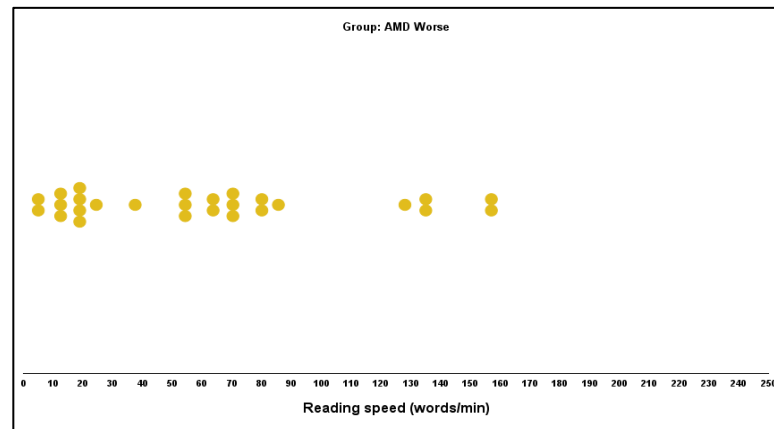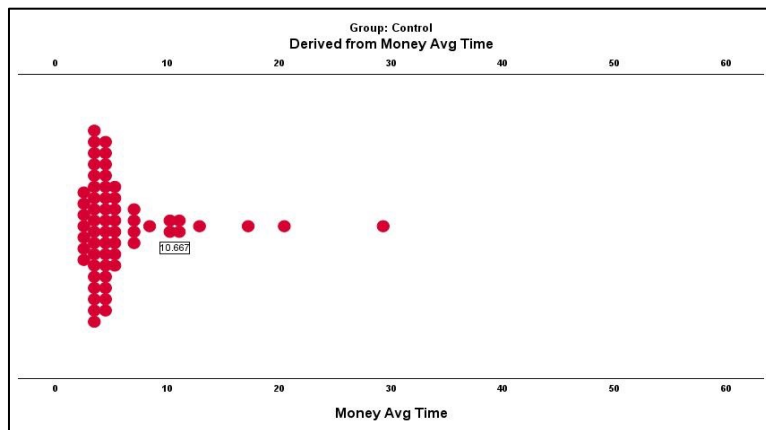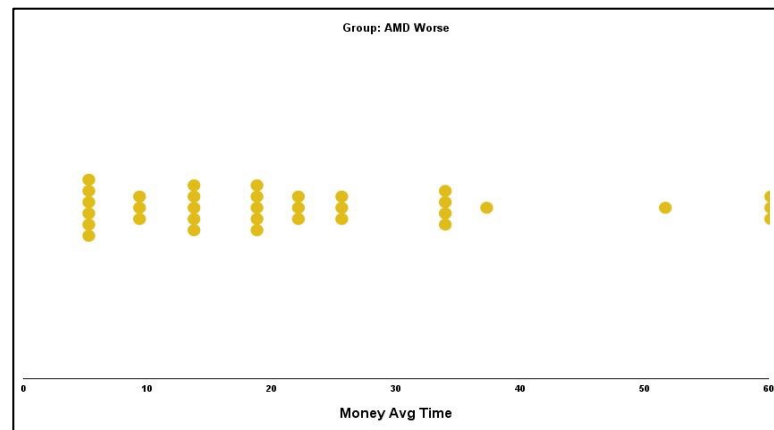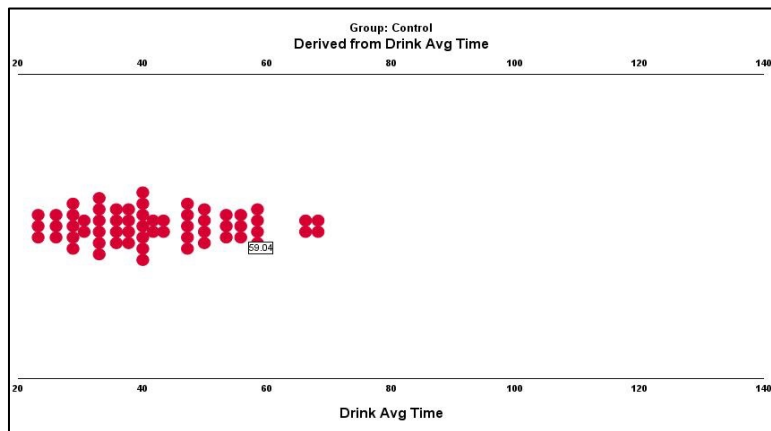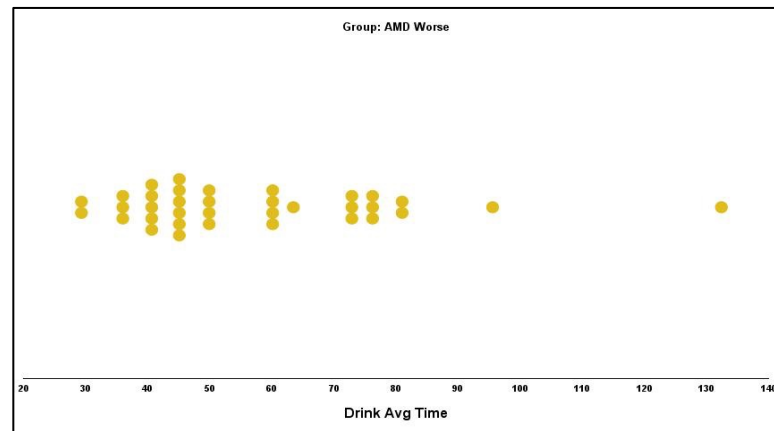

Supplement: Supplement 2 [file tvst-13-6-9_s002.pdf]
