# Supplementary material for: Development and Validation of Performance-Based Assessment of Daily Living Tasks in Age-Related Macular Degeneration
Source: Transl Vis Sci Technol. 2024 Jun 17;13(6):9. doi: 10.1167/tvst.13.6.9 (PMC11185266; doi:10.1167/tvst.13.6.9)
Supplement: Supplement 3 [file tvst-13-6-9_s003.pdf]

Supplementary table 1: Occupational therapist designed grading scale for ability to perform ADLTT (4) and (5) on the breakdown of the various tasks into steps

Scoring for money management and making a drink

0: dependent on others to perform the task
1: performs task with difficulty even under optimal conditions (using compensatory touch/visual strategies) (examples: difficulty performing task in a timely manner, questionable safety and accuracy)
2: performs task with no difficulty under optimal conditions (using compensatory touch/visual strategies)
3: independent: experience no difficulty performing tasks safely, accurately and efficiently

Grading breakdown of money counting tasks (Maximum score: 9)

1. Recognize coins
2. Locate coins
3. Count money

Grading breakdown of making drink task (Maximum score: 15)

1. Locate items
2. Cut/Open drink sachet
3. Pour drink powder
4. Pour liquid
5. Stir drink
