# Supplementary material for: Development and Validation of Performance-Based Assessment of Daily Living Tasks in Age-Related Macular Degeneration
Source: Transl Vis Sci Technol. 2024 Jun 17;13(6):9. doi: 10.1167/tvst.13.6.9 (PMC11185266; doi:10.1167/tvst.13.6.9)
Supplement: Supplement 4 [file tvst-13-6-9_s004.pdf]

Supplementary Table 2: Baseline characteristics of persons with age-related macular degeneration (AMD) versus normal controls

Baseline demographics	AMD (N=36)	Controls (N=36)	P value *
**Age (SD)**	72.1 ± 7.6	65.2 ± 7.7	<0.001
**Male (%)**	52.5%	55.0%	1.00
**Race n(%)**			0.10
• Chinese	33 (91.7%)	29 (80.6%)	
• Malay	2 (5.6%)	2 (5.6%)	
• Indian	1 (2.8%)	4 (11.1%)	
• Others	0	1 (2.8%)	
**Unaided visual acuity (logMAR± SD)**			
o Worse eye vision	1.15 ± 0.54	0.24 ± 0.21	<0.001
o Better eye vision	0.35 ± 0.23	0.14 ± 0.15	<0.001
o Binocular vision	0.27 ± 0.18	0.09 ± 0.09	<0.001
**Best-corrected visual acuity (logMAR ± SD)**			
o Worse eye vision	1.01 ± 0.60	0.08 ± 0.10	<0.001
o Better eye vision	0.23 ± 0.20	0.02 ± 0.05	<0.001
o Binocular vision	0.17 ± 0.16	0.01 ± 01.06	<0.001
**Contrast sensitivity (log ± SD)**			
o Worse eye vision	0.82 ± 0.50	1.59 ± 0.11	<0.001
o Better eye vision	1.44 ± 0.26	1.60 ± 0.09	0.001
o Binocular vision	1.49 ± 0.21	1.68 ± 0.04	<0.001
**MICROPERIMETRY RESULTS**			
**Average retinal sensitivity threshold (dB± SD)**			
• Worse eye vision	10.8 ± 7.6	27.2 ± 4.5	<0.001
• Better eye vision	20.0 ± 5.8	27.6 ± 4.8	<0.001
**Average macula sensitivity (dB± SD)**			
o Worse eye vision	5.0 ± 8.2	24.5 ± 6.5	<0.001
o Better eye vision	16.4 ± 7.3	25.4 ± 5.9	<0.001
**Time taken to complete microperimetry (s ± SD)**			
o Worse eye vision	495 ± 277	310 ± 177	0.002
o Better eye vision	383 ± 80	315 ± 207	0.079
**Fixation - Stable (%)**			
o Worse eye vision	7/36(19.4%)	21/34(64.8%)	<0.001
o Better eye vision	13/36(36.1%)	21/34(64.8%)	0.038

Worse eye and better eye vision defined based on best corrected visual acuity,

**p-value calculated by Chi-squared probability tests for categorical parameters and student's t-test for quantitative variables*
