# Supplementary material for: Development and Validation of Performance-Based Assessment of Daily Living Tasks in Age-Related Macular Degeneration
Source: Transl Vis Sci Technol. 2024 Jun 17;13(6):9. doi: 10.1167/tvst.13.6.9 (PMC11185266; doi:10.1167/tvst.13.6.9)
Supplement: Supplement 5 [file tvst-13-6-9_s005.pdf]

Supplementary table 3: Data supporting content validity of various ADLTTs were performed in their daily life as reported by the participants reported as mean scores.

Questions for participants	AMD (%) (N=33)	Controls (%) (N=35)	Total (%) (N=68)
Rate in terms of how important these tasks us for you daily life<br>1 (not important) --5 (moderately important) --10 (extremely important)			
		Mean scores (SD)	
• Reading	7.58 (3.0)	8.79(2.2)	8.16(2.7)
• Recognizing faces and expressions	7.25(2.7)	8.36(2.1)	7.78(2.5)
• Searching for items on a table	8.14(2.4)	8.27(2.7)	8.20(2.5)
• Counting money (coins)	8.50(2.3)	8.97(1.8)	8.72(2.1)
• Making a drink	9.11(2.0)	7.67(2.9)	8.42(2.6)
Rate how well you feel you can perform these following tasks with no help from others<br>1 (cannot perform) ------5 (can performed with some help) ------10 (performed well with no help)			
		Mean scores (SD)	
• Reading	8.83(2.8)	10.00(0)	9.39(2.1)
• Recognizing faces and expressions	9.39(1.8)	10.00(0)	9.68(1.3)
• Searching for items on a table	9.50(1.4)	10.00(0)	9.74(1.1)
• Counting money (coins)	9.44(1.6)	10.00(0)	9.71(1.2)
• Making a drink	9.72(1.2)	10.00(0)	9.86(0.9)
