# Supplementary material for: Development and Validation of Performance-Based Assessment of Daily Living Tasks in Age-Related Macular Degeneration
Source: Transl Vis Sci Technol. 2024 Jun 17;13(6):9. doi: 10.1167/tvst.13.6.9 (PMC11185266; doi:10.1167/tvst.13.6.9)
Supplement: Supplement 6 [file tvst-13-6-9_s006.pdf]

Supplementary table 4: The assessment of convergent validity though the correlation of binocular and monocular performance based ADLTT with patient reported outcome measures (visual function domain Rasch scores from the Impact of Visual Impairment Scale)

C=control; A=AMD;

		Binocular		Monocular	
Task		**Visual function domain (Rasch scores)**	p-value	**Visual function domain (Rasch scores)**	p-value
1) Reading speed (words/min)	C	0.08	0.66	0.04	0.71
	A	0.19	0.29	0.15	0.26
2) Number of facial expressions identified	C	0.07	0.69	-0.08	0.52
	A	0.24	0.17	0.06	0.65
3) Time taken to complete the item search task (s)	C	-0.10	0.56	-0.25	0.04
	A	0.14	0.43	0.18	0.18
4) Time taken to complete the money counting task (s)	C	-0.12	0.50	-0.16	0.18
	A	-0.06	0.72	0.11	0.43
5) Time taken to complete the making drink task (s)	C	0.02	0.92	-0.07	0.55
	A	0.13	0.47	0.05	0.70

†Monocular values from better and worse eyes combined for correlation analysis,
A=AMD group C = controls
Spearman correlation coefficient categories: poor=<0.3, fair= 0.3-0.49, moderately strong=0.6-0.79, very strong 0.80-1.0
(Reference Chan YH. Biostatistics 104: correlational analysis. Singapore Med J. 2003 Dec;44(12):614-9. PMID: 14770254)
