# Supplementary material for: Development and Validation of Performance-Based Assessment of Daily Living Tasks in Age-Related Macular Degeneration
Source: Transl Vis Sci Technol. 2024 Jun 17;13(6):9. doi: 10.1167/tvst.13.6.9 (PMC11185266; doi:10.1167/tvst.13.6.9)
Supplement: Supplement 7 [file tvst-13-6-9_s007.pdf]

Supplementary Table 5: The assessment of criterion validity in performance-based measurement of the activities of daily living tasks tests (ADLTT) performed with monocular vision in eyes based on visual acuity

ADLTT (monocular scores only) (N=no of eyes)	Good VA logMAR<0.3	Moderate VA 0.3≤logMAR<0.7	Poor VA (logMAR>0.7)	**p-value
**1. Reading tests** **Speed (words/min) (SD)**				
○ Monocular	132.1(59.7) (N=124)	117.5(39.8) (N=20)	78.7(82.3) (N=18)	0.065
○ Binocular	148.5(36.8) (N=87)	95.2(44.1) (N=16)	NA	<0.001
**2. Facial Expression#** **Mean number of expressions identified (max score =5)(SD)**				
○ Monocular	3.2(1.3) (N=128)	3.8(0.9) (N=16)	4.3(1.9) (N=18)	0.128
○ Binocular	3.7(1.1) (N=87)	3.7(1.0) (N=21)	NA	0.970
**3. Item search task ^** **Time taken to complete task (s)(SD)**				
○ Monocular	10.2(8.1) (N=126)	10.3(5.6) (N=16)	5.6(10.7) (N=26)	0.102
○ Binocular	7.5(5.6) (N=87)	7.6(6.4) (N=21)	NA	0.934
**Number of items identified correctly (max score=4)(SD)**				
○ Monocular	3.8(1.6) (N=126)	3.77(0.3) (N=16)	3.9(0.6) (N=26)	0.296
○ Binocular	3.9(1.5) (N=87)	3.7(0.2) (N=21)	NA	0.257
**4. Money counting task ^** **Time taken to complete task (s)(SD)**				
○ Monocular	8.3(1.6) (N=126)	12.4(10.5) (N=16)	20.1(2.4) (N=24)	0.155
○ Binocular	5.7(4.7) (n=87)	9.9(11.6) (N=87)	NA	0.157
**Ability of task completion (max score=9) (SD)**				
○ Monocular	8.3(1.5) (N=126)	8.9(1.1) (N=16)	8.8(2.4) (N=24)	0.263
○ Binocular	8.9(0.3) (N=87)	8.87(0.5) (N=21)	NA	0.627
**5. Making drink task ^** **Time taken to complete task (s)(SD)**				
○ Monocular	48.9(23.3) (N=126)	46.7(13.3) (N=16)	51.2(22.2) (N=25)	
○ Binocular	44.6(29.2) (N=87)	44.4(26.8) (N=21)	NA	
**Ability of task completion (max score =9)(SD)**				
○ Monocular	14.7(1.5) (N=126)	14.7(1.2) (N=16)	14.2(2.0) (N=25)	0.579
○ Binocular	14.98(0.1) (N=87)	14.8(0.9) (N=21)	NA	0.340

**p-value calculated by ANOVA for quantitative variables # Mean score taken over 3 attempts ^Mean values taken over 2 attempts

Monocular series :Linear Mixed Model Binocular series: GEE used as Mixed Model has Hessian Matrix convergence issues.
