# Supplementary material for: Development and Validation of Performance-Based Assessment of Daily Living Tasks in Age-Related Macular Degeneration
Source: Transl Vis Sci Technol. 2024 Jun 17;13(6):9. doi: 10.1167/tvst.13.6.9 (PMC11185266; doi:10.1167/tvst.13.6.9)
Supplement: Supplement 8 [file tvst-13-6-9_s008.pdf]

Supplementary table 6: The availability of the assessment of the psychometric properties of the ADLTTs that was performed in our validation study based on the modified COSMIN checklist for PCOMs.

ADLTT	PCOM development	Content validity	Cross-cultural validity	Reliability/ Measurement error	Criterion validity	Convergent/ Known group validity
Reading test (IREST)	Very good	Adequate	Adequate	Adequate	Very good	Very good
Facial expression	Very good	Adequate	Adequate	Adequate	N/A	Very good
Item search	Very good	Adequate	Adequate	Adequate	N/A	Very good
Money counting	Very good	Adequate	Adequate	Adequate	N/A	Very good
Making drink	Very good	Adequate	Adequate	Adequate	N/A	Very good
