# Supplementary material for: Development and Validation of Performance-Based Assessment of Daily Living Tasks in Age-Related Macular Degeneration
Source: Transl Vis Sci Technol. 2024 Jun 17;13(6):9. doi: 10.1167/tvst.13.6.9 (PMC11185266; doi:10.1167/tvst.13.6.9)
Supplement: Supplement 9 [file tvst-13-6-9_s009.docx]

COSMIN Risk of Bias checklist (items relevant to patient-centred performance-based outcome measures)

**Date:** December 2023

*How to site the COSMIN Risk of Bias Checklist*

Please refer to the following studies when using the COSMIN Risk of Bias Checklist:

Mokkink, L.B., De Vet, H.C.W., Prinsen, C.A.C, Patrick, D.L., Alonso, J., Bouter, L.M., et al. COSMIN Risk of Bias checklist for systematic reviews of Patient‐Reported Outcome Measures. Accepted for publication in Quality of Life Research.

Prinsen, C. A., Mokkink, L. B., Bouter, L. M., Alonso, J., Patrick, D. L., Vet, H. C., et al. COSMIN guideline for systematic reviews of Patient‐Reported Outcome Measures. Submitted.

Terwee, C. B., Prinsen, C. A., Chiarotto, A., Vet, H. C., Westerman, M. J., Patrick, D. L., et al. COSMIN methodology for evaluating the content validity of Patient‐Reported Outcome Measures: a Delphi study. Submitted.

For details on how to use the COSMIN risk of Bias checklist see ‘COSMIN methodology for systematic reviews of Patient‐Reported Outcome Measures (PROMs) – user manual’ and ‘COSMIN methodology for assessing the content validity of Patient‐Reported Outcome Measures (PROMs) ‐ user manual’ available from our website [www.cosmin.nl.](http://www.cosmin.nl/)

*Abbreviations used:*

*CTT – classical test theory*

*DIF – differential item functioning IRT – Item response theory*

*MGCFA – multi‐group confirmatory factor analysis MI – measurement invariance*

*NA – not applicable*

*PROM – patient‐reported outcome measure 1PL model – 1 parameter IRT model*

*2PL model – 2 parameter IRT model*

**Instructions**

*Tick the boxes that need to be completed for the article*

|  | **COSMIN Risk of Bias checklist** |
| --- | --- |
| X | Box 1. PROM development |
| X | Box 2. Content validity |
|  | Box 3. Structural validity |
|  | Box 4. Internal consistency |
| X | Box 5. Cross‐cultural validity\Measurement invariance |
| X | Box 6. Reliability |
| X | Box 7. Measurement error |
| X | Box 8. Criterion validity |
| X | Box 9. Hypotheses testing for construct validity |
| X | Box 10. Responsiveness |

To assess the methodological quality of each study, i.e. assessing the risk of bias of the result of a study, the corresponding COSMIN Risk of Bias box should be completed. To determine the overall quality of a study the lowest rating of any standard in the box is taken (i.e. “the worst score counts” principle). For example, if for a reliability study one item in a box is rated as ‘inadequate’, the overall methodological quality of that reliability study is rated as ‘inadequate’. The response option ‘NA’ (not applicable) is at issue for some standards. For example, when a study on structural validity is based on CTT, the standard on IRT is not applicable and this standard should not be considered in the “worst score counts”‐ rating for that specific study. For standards where this option is not at issue, these cells are grey and shouldn’t be used.

| **Box 1. PCOM development 1a. PCOM design** | | | | | |
| --- | --- | --- | --- | --- | --- |
| *General design requirements* | **very good** | **adequate** | **doubtful** | **inadequate** | **NA** |
| 1 Is a clear description provided of the construct to be measured? | Construct clearly described | Assumable that the study was performed in a sample representing the target population, but not clearly  described |  | Construct not clearly  described |  |
| 2 Is the origin of the construct clear: was a theory, conceptual  framework or disease model used or clear rationale provided to define the construct to be measured? | Origin of the  construct clear |  | Origin of the construct  not clear |  |  |
| 3 Is a clear description provided of the target population for which the PCOM was developed? | Target population clearly described |  |  | Target population not clearly  described |  |
| 4 Is a clear description provided of the context of use | Context of use  clearly described |  | Context of use not  clearly described |  |  |
| 5 Was the PCOM development study performed in a sample representing the target population for which the PCOM was developed? | Study performed in a sample representing the target population |  | Doubtful whether the study was performed in a sample representing the target population | Study not performed in a sample representing the target population **(SKIP items 6‐12)** |  |

| **Box 2. Content validity**  **2a. Asking patients about relevance** | | | | | | |
| --- | --- | --- | --- | --- | --- | --- |
| *Design requirements* | | **very good** | **adequate** | **doubtful** | **inadequate** | **NA** |
| 1 | Was an appropriate method used to ask patients whether the PCOM is relevant for their experience with the condition?  Was each item tested in an appropriate number of patients?  For qualitative studies  Were skilled group moderators/interviewers used?  Were the group meetings or interviews based on an appropriate topic or interview guide? | Widely recognized or well justified method used | Only quantitative (survey) method(s) used or assumable that the method was appropriate but not clearly  described | Not clear if patients were asked whether each item is relevant or doubtful whether the method was appropriate | Method used not appropriate or patients not asked about the relevance of all items |  |
| 2 |  |  |  |  |  |  |
|  |  | ≥7 | 4‐6 | <4 or not clear |  |  |
| 3 |  | Skilled group moderators/ interviewers used | Group moderators  /interviewers had limited experience or were trained specifically for the study | Not clear if group moderators  /interviewers were trained or group moderators  /interviewers not trained and no experience |  | Not applicable |
| 4 |  | Appropriate topic or interview guide | Assumable that the topic or interview guide was appropriate, but not clearly described | Not clear if a topic guide was used or doubtful if topic or interview guide was appropriate or no guide |  | Not applicable |

| 5 Were the group meetings or interviews recorded and transcribed verbatim? | All group meetings or interviews were recorded and transcribed verbatim | Assumable that all group meetings or interviews were recorded and transcribed verbatim, but not clearly described | Not clear if all group meetings or interviews were recorded and transcribed verbatim or recordings not transcribed verbatim or only notes were made during the group meetings/ interviews | No recording and no notes | Not applicable |
| --- | --- | --- | --- | --- | --- |
| *Analyses* |  |  |  |  |  |
| 6 Was an appropriate approach used to analyse the data? | A widely recognized or well justified approach was used | Assumable that the approach was appropriate, but not clearly described | Not clear what approach was used or doubtful whether the approach was appropriate | Approach not appropriate |  |
| 7 Were at least two researchers involved in the analysis? | At least two researchers involved in the analysis | Assumable that at least two researchers were involved in the analysis, but not clearly described | Not clear if two researchers were included in the analysis or only one researcher involved in the analysis |  |  |

| **2d. Asking professionals about relevance** | | | | |
| --- | --- | --- | --- | --- |
| *Design requirements* | **very good** | **adequate** | **doubtful inadequate** | **NA** |
| 1. Was an appropriate method used to ask professionals whether each item is relevant for the construct of interest? 2. Were professionals from all relevant disciplines included? 3. Was each item tested in an appropriate number of professionals? For qualitative studies   *Analyses*   1. Was an appropriate approach used to analyse the data? | Widely recognized or well justified method used | Only quantitative (survey) method(s) used or assumable that the method was appropriate but not clearly described | Not clear if Method used professionals were not  asked whether appropriate or each item is professionals  relevant or not asked doubtful whether about the  the method was relevance of all appropriate items | |
|  | Professionals from all required disciplines were included | Assumable that professionals from all required disciplines were included, but not clearly described | Doubtful whether professionals from all required disciplines were included or relevant professionals were not included | |
|  | ≥7 | 4‐6 | <4 or not clear | |
|  | A widely recognized or well justified approach was used | Assumable that the approach was appropriate, but not clearly described | Not clear what Approach not approach was used appropriate or doubtful  whether the approach was appropriate | |

| 26 Were at least two researchers involved in the analysis? | At least two researchers Assumable that at Not clear if two involved in the analysis least two researchers were  researchers were included in the involved in the analysis or only analysis, but not one researcher clearly described involved in the  analysis |  |
| --- | --- | --- |

| **Box 5. Cross‐cultural validity\Measurement invariance** | | | | | |
| --- | --- | --- | --- | --- | --- |
| *Design requirements* | **very good** | **adequate** | **doubtful** | **inadequate** | **NA** |
| 1 Were the samples similar for relevant characteristics except for the group variable? | Evidence provided that samples were similar for relevant characteristics except group variable | Stated (but no evidence provided) that samples were similar for relevant characteristics except group variable | Unclear whether samples were similar for relevant characteristics except group variable | Samples were NOT similar for relevant characteristics except group variable |  |
| *Statistical methods* |  |  |  |  |  |
| 2 Was an appropriate approach used to analyse the data? | A widely recognized or well justified approach was used | Assumable that the approach was appropriate, but not clearly described | Not clear what approach was used or doubtful whether the approach was appropriate | Approach not appropriate | Not applicable |
| 3 Was the sample size included in the analysis adequate? | Regression analyses or IRT/Rasch based analyses: 200 subjects per group | 150 subjects per group | 100 subjects per group | < 100 subjects per group |  |
|  | MGCFA*: 7 times the number of items and ≥100 | 5 times the number of items and ≥100; OR 5‐7 times the number of items but <100 | 5 times the number of items but <100 | <5 times the number of items |  |
| *Other* |  |  |  |  |  |
| 4 Were there any other important flaws in the design or statistical methods of the study? | No other important methodological  flaws |  | Other minor methodological flaws | Other important methodological  flaws |  |

**MGCFA: multi‐group confirmatory factor analyses*

| **Box 6. Reliability** | | | | | |
| --- | --- | --- | --- | --- | --- |
| *Design requirements* | **very good** | **adequate** | **doubtful** | **inadequate** | **NA** |
| 1 Were patients stable in the interim period on the construct to be measured? | Evidence provided that patients were stable | Assumable that patients were stable | Unclear if patients were stable | Patients were NOT stable |  |
| 2 Was the time interval appropriate? | Time interval appropriate |  | Doubtful whether time interval was appropriate or time interval was not stated | Time interval NOT  appropriate |  |
| 3 Were the test conditions similar for the measurements? e.g. type of administration, environment, instructions | Test conditions were similar (evidence provided) | Assumable that test conditions were similar | Unclear if test conditions were similar | Test conditions were NOT similar |  |
| *Statistical methods* |  |  |  |  |  |
| 1. For continuous scores: Was an intraclass correlation coefficient (ICC) calculated? 2. For dichotomous/nominal/ordinal scores: Was kappa calculated? | ICC calculated and model or formula of the ICC is described  Kappa calculated | ICC calculated but model or formula of the ICC not described or not optimal.  Pearson or Spearman correlation coefficient calculated with evidence provided that no systematic change has occurred | Pearson or Spearman correlation coefficient calculated WITHOUT evidence provided that no systematic change has occurred or WITH evidence that systematic change has occurred | No ICC or Pearson or Spearman correlations calculated  No kappa calculated | Not applicable  Not applicable |

| 6 | For ordinal scores: Was a weighted kappa calculated? | Weighted Kappa calculated |  | Unweighted Kappa calculated or not  described |  | Not applicable |
| --- | --- | --- | --- | --- | --- | --- |
| 7 | For ordinal scores: Was the weighting scheme described? e.g.  linear, quadratic | Weighting scheme  described | Weighting scheme  NOT described |  |  | Not  applicable |
| *Other* | |  |  | |  | |
| 8 | Were there any other important flaws in the design or statistical | No other | Other minor | | Other | |
|  | methods of the study? | important | methodological flaws | | important | |
|  |  | methodological |  | | methodological | |
|  |  | flaws |  | | flaws | |

| **Box 7. Measurement error** | | | | | |
| --- | --- | --- | --- | --- | --- |
| *Design requirements* | **very good** | **adequate** | **doubtful** | **Inadequate** | **NA** |
| 1 Were patients stable in the interim period on the construct to be measured? | Patients were stable (evidence provided) | Assumable that patients were stable | Unclear if patients were stable | Patients were NOT stable |  |
| 2 Was the time interval appropriate? | Time interval appropriate |  | Doubtful whether time interval was appropriate or time interval was not stated | Time interval NOT  appropriate |  |
| 3 Were the test conditions similar for the measurements? (e.g. type of administration, environment, instructions) | Test conditions were similar (evidence provided) | Assumable that test conditions were similar | Unclear if test conditions were similar | Test conditions were NOT similar |  |
| *Statistical methods* |  |  |  |  |  |
| 1. For continuous scores: Was the Standard Error of Measurement (SEM), Smallest Detectable Change (SDC) or Limits of Agreement (LoA) calculated? 2. For dichotomous/nominal/ordinal scores: Was the percentage (positive and negative) agreement calculated? | SEM, SDC, or LoA  calculated  % positive and negative agreement  calculated | Possible to calculate LoA from the data presented  % agreement calculated |  | SEM calculated based on Cronbach’s alpha, or on SD from another population  % agreement not calculated | Not applicable  Not applicable |
| *Other* |  |  |  |  |  |
| 6 Were there any other important flaws in the design or statistical methods of the study? | No other important methodological flaws |  | Other minor methodological flaws | Other important methodological  flaws |  |

| **Box 8. Criterion validity** | | | | | |
| --- | --- | --- | --- | --- | --- |
|  | **very good** | **adequate** | **doubtful** | **inadequate** | **NA** |
| *Statistical methods* |  |  |  |  |  |
| 1 For continuous scores: Were correlations, or the area under the receiver operating curve calculated? | Correlations or AUC calculated |  |  | Correlations or AUC NOT  calculated | Not applicable |
| 2 For dichotomous scores: Were sensitivity and specificity determined? | Sensitivity and specificity calculated |  |  | Sensitivity and specificity NOT  calculated | Not applicable |
| *Other* |  |  |  |  |  |
| 3 Were there any other important flaws in the design or statistical methods of the study? | No other important methodological  flaws |  | Other minor methodological  flaws | Other important methodological  flaws |  |

| **Box 9. Hypotheses testing for construct validity**  **9a. Comparison with other outcome measurement instruments (convergent validity)** | | | | | |
| --- | --- | --- | --- | --- | --- |
| *Design requirements* | **very good** | **adequate** | **doubtful** | **inadequate** | **NA** |
|  |  | | | | |
| 1 Is it clear what the comparator instrument(s) measure(s)? | Constructs measured by the comparator instrument(s) is  clear |  | | Constructs measured by the comparator instrument(s) is  not clear |  |
| 2 Were the measurement properties of the comparator instrument(s) sufficient? | Sufficient measurement properties of the comparator instrument(s) in a population similar to the study population | Sufficient measurement properties of the comparator instrument(s) but not sure if these apply to the study population | Some information on measurement properties of the comparator instrument(s) in any study population | No information on the measurement properties of the comparator instrument(s), OR evidence for insufficient measurement properties of the comparator  instrument(s) |  |
| *Statistical methods* |  | | | | |
| 3 Was the statistical method appropriate for the hypotheses to be tested? | Statistical method was appropriate | Assumable that statistical method was appropriate | Statistical method applied NOT optimal | Statistical method applied NOT appropriate |  |

| *Other* |  | | | |
| --- | --- | --- | --- | --- |
| 4 Were there any other important flaws in the design or statistical methods of the study? | No other important methodological flaws |  | Other minor Other important methodological flaws methodological (e.g. only data flaws  presented on a comparison with an instrument that measures another  construct) |  |

**9b. Comparison between subgroups (discriminative or known‐groups validity)**

*Design requirements*

Adequate

description of the important characteristics of the subgroups

Adequate

description of most of the important

Poor of no description

of the important characteristics of the

characteristics of the subgroups

subgroups

Statistical method Assumable that Statistical method Statistical method

was appropriate statistical method applied NOT optimal applied NOT was appropriate appropriate

No other important

methodological flaws

Other minor Other important

methodological flaws methodological (e.g. only data flaws presented on a

comparison with an instrument that measures another construct)

7 Were there any other important flaws in the design or statistical

methods of the study?

*Statistical methods*

6 Was the statistical method appropriate for the hypotheses to be tested?

*Other*

5 Was an adequate description provided of important characteristics

of the subgroups?

**NA**

**inadequate**

**doubtful**

**adequate**

**very good**

| **Box 10. Responsiveness**  **10a. Criterion approach (i.e. comparison to a gold standard)** | | | | | |
| --- | --- | --- | --- | --- | --- |
|  | **very good** | **adequate** | **doubtful** | **inadequate** | **NA** |
| *Statistical methods* |  |  |  |  |  |
| 1 For continuous scores: Were correlations between change scores, or the area under the Receiver Operator Curve (ROC) curve calculated? | Correlations or Area under the ROC Curve (AUC) calculated |  |  | Correlations or AUC NOT  calculated | Not applic able |
| 2 For dichotomous scales: Were sensitivity and specificity (changed versus not changed) determined? | Sensitivity and specificity calculated |  |  | Sensitivity and specificity NOT calculated | Not applic able |
| *Other* |  |  |  |  |  |
| 3 Were there any other important flaws in the design or statistical methods of the study? | No other important methodological  flaws |  | Other minor Other important methodological flaws methodological  flaws | |  |

| **10b. Construct approach (i.e. hypotheses testing; comparison with other outcome measurement instruments)** | | | | | | |
| --- | --- | --- | --- | --- | --- | --- |
| *Design requirements* | | **very good** | **adequate** | **doubtful** | **inadequate** | **NA** |
|  |  |  | | | | |
| 4 | Is it clear what the comparator instrument(s) measure(s)? | Constructs measured by the comparator instrument(s) is clear |  | | Constructs measured by the comparator instrument(s) is not clear |  |
| 5 | Were the measurement properties of the comparator instrument(s) sufficient? | Sufficient measurement properties of the comparator instrument(s) in a population similar to the study population | Sufficient measurement properties of the comparator instrument(s) but not sure if these apply to the study population | Some information on measurement properties of the comparator instrument(s) in any study population | NO information on the measurement properties of the comparator instrument(s) OR evidence of poor quality of comparator  instrument(s) |  |
| *Statistical methods* | |  | | | | |
| 6 | Was the statistical method appropriate for the hypotheses to be tested? | Statistical method was appropriate | Assumable that statistical method  were appropriate | Statistical method applied NOT optimal | Statistical method applied NOT  appropriate |  |
| *Other* | |  | | | | |
| 7 | Were there any other important flaws in the design or statistical methods of the study? | No other important methodological  flaws |  | Other minor Other important methodological flaws methodological  flaws | |  |

| **10c. Construct approach: (i.e. hypotheses testing: comparison between subgroups)** | | | | | |
| --- | --- | --- | --- | --- | --- |
| *Design requirements* | **very good** | **adequate** | **doubtful** | **inadequate** | **NA** |
|  |  | | | | |
| 8 Was an adequate description provided of important characteristics of the subgroups? | Adequate description of the important characteristics of the  subgroups | Adequate description of most of the important characteristics of the  subgroups | Poor or no description of the important characteristics of the subgroups |  | |
| *Statistical methods* |  | | | | |
| 9 Was the statistical method appropriate for the hypotheses to be tested? | Statistical method was appropriate | Assumable that statistical method  was appropriate | Statistical method applied NOT optimal | Statistical method applied NOT  appropriate |  |
| *Other* |  | | | | |
| 10 Were there any other important flaws in the design or statistical methods of the study? | No other important methodological  flaws |  | Other minor methodological flaws | Other important methodological  flaws |  |

| **10d. Construct approach: (i.e. hypotheses testing: before and after intervention)** | | | | | |
| --- | --- | --- | --- | --- | --- |
| *Design requirements* | **very good** | **adequate** | **doubtful** | **inadequate** | **NA** |
|  |  | | | | |
| 11 Was an adequate description provided of the intervention given? | Adequate description of the intervention |  | Poor description of the intervention | NO description of the intervention |  |
| *Statistical methods* |  | | | | |
| 12 Was the statistical method appropriate for the hypotheses to be tested? | Statistical method was appropriate | Assumable that statistical method  was appropriate | Statistical method applied NOT optimal | Statistical method applied NOT  appropriate |  |
| *Other* |  | | | | |
| 13 Were there any other important flaws in the design or statistical methods of the study? | No other important methodological  flaws |  | Other minor methodological flaws | Other important methodological  flaws |  |
